# Anti-inflammatory activity of nicotine isolated from *Brassica oleracea* in rheumatoid arthritis

**DOI:** 10.1042/BSR20211392

**Published:** 2022-04-21

**Authors:** Bharti Rakhecha, Prachi Agnihotri, Tikam Chand Dakal, Mohd Saquib, Monu, Sagarika Biswas

**Affiliations:** 1 Council of Industrial Research (CSIR)-Institute of Genomics and Integrative Biology, Mall Road, Delhi University Campus, Delhi 110007, India; 2 Academy of Scientific and Innovative Research (AcSIR), Ghaziabad 201002, India; 3 Mohanlal Sukhadia University, Udaipur, Rajasthan 313001, India

**Keywords:** HPLC, Nicotine, Rheumatoid arthritis, SW982 cells, TNFα

## Abstract

**Objectives:** Rheumatoid arthritis (RA) is an autoimmune disease, associated with chronic inflammation of synoviocytes. Tumor necrosis factor α (TNF-α) plays a crucial role in the pathogenesis of RA through pro-inflammatory cytokines. Nicotine, an alkaloid used as herbal medicine, often worked as an anti-inflammatory agent. In the present study, we tried to uncover the anti-inflammatory impact of nicotine against RA.

**Materials and methods:** Nicotine was isolated from *Brassica oleracea*, purified by high profile/phase liquid chromatography (HPLC). *In-silico* docking was carried out using bioinformatics tools SwissADME (absorption, distribution, metabolism and excretion), PASS, and Drug-induced Gene Expression Profile (DIGEP)-Pred to determine drug likeliness of nicotine. The *in-vitro* study was performed in TNFα-induced SW982 synoviocytes by qPCR. mRNA expression of pro-inflammatory cytokines (TNF, IL6, IL1β) and proteins (TRAF2, P50, P65) were analyzed followed by validation of P65 (RELA), pP65, IkBα by Western blot analysis.

**Results:** Nicotine compound was extracted from *Brassica oleracea* and purified by HPLC method (R_t_ values at 2.67 min). The physicochemical, pharmacokinetic properties and drug-likeliness of nicotine were studied by *in-silico* analysis. *In-vitro* studies revealed that nicotine lowers the expression of inflammatory cytokines (TNF, IL6, IL1β) and proteins (TNF receptor-associated factor 2 (TRAF2), P50, P65) at 1 µg/ml in TNFα-induced SW982 cells.

**Conclusion:** Nicotine from natural sources (*Brassica oleracea*) has been found to be an effective anti- inflammatory compound at a low dosage; thus, identifying the role of nicotine present in the natural sources as a therapeutic option for RA, may be recommended as remedial drug instead of synthetic drug.

## Introduction

Rheumatoid arthritis (RA) is a systemic inflammation disorder, classified as a multifactorial autoimmune disease, known to associate with complex etiologic factors [1]. Approximately 0.5–1.0% of the world population is reported to be affected by RA with women being affected three-times (0.5–3.8%) more than men (0.15–1.37%) [2]. In India, less than 1% population is affected by RA with increasing age, maximum affected population is between 35 and 50 years of age [3]. RA is characterized by inflammation of the synovial tissue, painful and swollen joints leading to significant disabilities [1]. T cells, B cells, and the complex interaction between receptors and pro-inflammatory cytokines also play key roles in the pathophysiology of RA [4]. It, thus, leads to persistent inflammation of synovial tissues, resulting into irreversible damage to the arthrodial joints and other associated body parts. Currently available drugs such as corticosteroids, non-steroidal anti-inflammatory drugs (NSAIDs) and disease-modifying anti-rheumatic drugs (DMARDs) are employed in treating RA, causing serious side effects [5]. The current therapeutic agents have thus fallen short in overcoming the RA inflammation without side effects.

RA progression involves interplaying between adaptive and innate immune system; their released cytokines and chemokines initiate the pathogenesis environment near synovium. Multiple types of immune cells get deposited near synovial membrane, leading to joint destruction [6]. Fibroblast-like synoviocytes (FLSs) are the permanent residence of synovium but their phenotype gets altered during inflammation in RA [7]. Tumor necrosis factor α (TNF-α) is the major cytokine, gets released by immune cells, is found in higher concentration in serum and synovial fluid, leading to inflammation in RA patients. Tumor necrosis factor α (TNFα) works synergistically with IL-7 resulting into defective activity of Tregs which in turn activates the release of osteoclastogenic cytokines by T cells, leading to bone destruction [7,8].

Therefore, an assessment of potentially therapeutic drugs offered by naturally occurring compounds are essentially required to be investigated for their potential sites of action as a therapeutic option of RA. Beneficiary effects of bioactive compounds extracted from various vegetables, mushrooms, fish, citrus fruits etc. have been reported earlier that aided in the betterment of the health conditions of RA patients [9,10]. *Brassica* vegetables suppress multiple etiological effects; oxidative stress, DNA damage, immune system stimulation, induction of detoxification mechanism through activation of enzymes and anti-inflammatory mechanism by activating various bioactive compounds [11]. The identification of dietary bioactive compounds/components from *Brassica* vegetables are therefore thought to be carried out with a belief that the identified compound might show an anti-inflammatory effect and may reduce RA pathogenesis.

In the present study, the role of nicotine isolated from *Brassica oleracea* extracts was investigated on TNFα-induced human synovial sarcoma cell line (SW982). Nicotine is a phytochemical present in various plant species of *Solanaceae* or nightshade family like *Nicotiana tabacum, Nicotiana glauca, Nicotiana sylvestris* and in lower quantities in tomato, potato, eggplant (aubergine) and cauliflower [12]. It is a natural tobacco alkaloid, comprising 95% total alkaloid compound, acts as an insecticide in tobacco leaves and consists of 1.5% in commercial tobacco cigarettes [13]. Our study revealed significant anti-inflammatory effect of nicotine at lower dose, inhibited TNF receptor-associated factor 2 (TRAF2) activation involved in nuclear factor-κ B (NF-kB) pathway. Our results thus triggered the studies on nicotine as a possible therapeutic agent for arthritis and other inflammatory diseases.

## Materials and methods

### Preparation of extracts

#### Sample preparation

Nicotine was extracted using cold extraction method using methanol solvent. Fresh *Brassica oleracea botrytis* (cauliflower) was purchased from a local market New Delhi, India. Head portion (edible part) was separated, rinsed, dried in hot air oven at 56°C, powdered, soaked in methanol for 48 h, then filtered through Whatman paper. The mixture was then subjected to high profile/phase liquid chromatography (HPLC) using HPLC-grade acetonitrile and methanol (Merck) [14]. In HPLC, methanol and acetonitrile (1:1) was used as mobile phase and each elution was checked between 0 and 30 min.

#### HPLC instrumentation and chromatographic condition

HPLC analysis was performed using an EZ Chrome Elite chromatography data system (Santa Clara, CA 95051, U.S.A.) with UV-VIS detector, EZ CHROME Software and an AGILENT C18 column (Poroshell 300SB-C182, 1 × 75 mm, 5 μm PN 660750-902, Santa Clara, CA 95051, U.S.A.).

A reverse-phase HPLC assay was carried out using an isocratic system with a flow rate of 1.0 ml/min, a column temperature of 25°C, a mobile phase of acetonitrile and methanol (50:50, v/v) and a detection wavelength of 265 nm [15]. The injection volume (50 μl) was filtered through a 0.2-μm nylon membrane prior to injection. The total chromatographic analysis time was 30 min per sample. Same conditions were applied for standard as well as for sample. Commercially available tobacco leaves extract was used as the standard.

### *In-silico* docking of nicotine with pro-inflammatory cytokine receptors

*In-silico* docking was carried out to find the interactions between nicotine and TNFR. Nicotine (Pubchem CID: 157672) was used as a ligand in mol2 format, TNFR(1EXT) was prepared for docking with an input in .pdb format in Swiss-Dock web server (http://www.swissdock.ch/) [16]. Prior to docking, protein structure was prepared by removing water molecules using UCSF Chimera software. The interaction of the compound with proteins and formation of hydrogen bond between the two molecules were visualized by Discovery Studio Visualizer (https://discover.3ds.com/discovery-studio-visualizer-download) and UCSF Chimera (https://www.cgl.ucsf.edu/chimera/).

### Drug-likeliness of nicotine

The drug-likeliness properties were analyzed using SwissADME (absorption, distribution, metabolism and excretion) tool. The Canonical simplified molecular input line-entry system (SMILES) format of the compound was retrieved by PubChem (https://pubchem.ncbi.nlm.nih.gov/) and submitted to SwissADME (http://www.swissadme.ch/) [17]. Drug-likeliness was determined by Lipinski’s rule of five (RO5) [18]. It can be characterized as a complex balance of various molecular properties such as absorption, distribution, metabolism, excretion and structural features such as molecular weight, number of hydrogen bond acceptor or donor, lipophilicity, molar refractivity that determines whether a molecule is a drug or not. Nicotine was thus subjected to various physicochemical and pharmacokinetic analyses.

#### Analysis of physiochemical and pharmacokinetics properties

Physicochemical and pharmacokinetic properties were analyzed using SwissADME (http://www.swissadme.ch/) [17]. The program was run using a molecule to be estimated as an input to Canonical SMILES.

#### Prediction of biological activity

Evaluation of the general biological potential of compound was carried out using PASS (http://195.178.207.233/PASS/index.html) based on their structural formula. The cut-off value Pa ≥ 0.7 was set and the only activity with Pa > Pi (Pa = probability ‘to be active’ and Pi = probability ‘to be inactive’) was considered. If Pa > 0.7, then that compound was predicted and selected for further studies [19].

#### Prediction of changes in gene expression

Drug-induced Gene Expression Profile (DIGEP)-Pred is a web service for *in-silico* prediction of drug-induced changes in gene expression profiles, based on structural formula of chemical compounds. The genes regulated by the compound—either up- or down-regulation can be studied based on protein prediction. The changes in gene expression profile were predicted by Pa (to be active) and Pi (to be inactive) values for each gene. The Pa and Pi values varied between 0 and 1. Parameters set were Pa > Pi and Pa > 0.5 [20].

### SW982 synovial sarcoma cell line culture

SW982 was obtained from National Centre for Cell Science (NCCS). The cells were cultured with Dulbecco’s modified Eagle’s medium (DMEM) supplemented with 10% fetal bovine serum and 1% antimycotic– antibacterial solution, incubated at 37°C with 5% CO_2_ [21].

### SW982 cells survivability assay

SW982 cells were seeded at concentrations of 1 × 10^4^ cells/well in 96-well culture plates. After 70–80% confluence, cells were treated with various concentrations of nicotine (1, 3, 5, 7 μg/ml) in serum-free media for 24 hrs. The cell viability was measured using 3-(4, 5-dimethylthiazol-2-yl)-2,5-diphenyl tetrazolium bromide (MTT) assay as per manufacturer’s instructions and absorbance was measured at 540 nm [21]. Cells treated with 20% dimethyl sulfoxide (DMSO) were used as a control.

### Cell survivability (Trypan Blue) staining

SW982 cells were seeded at concentrations of 1 × 10^4^ cells/well in 96-well culture plates. After 70–80% confluence, cells were treated with various concentrations of nicotine (1, 3, 5 μg/ml) in serum-free media for 24 h [21]. Cells were trypsinized and pellets were mixed in PBS, thoroughly mixed with 1:1 dilution Trypan Blue stain (10 µl of cells and 10 µl of 0.4% Trypan Blue stain) in vial. The mixture was incubated at room temperature (RT) for 5 min and then 20 µl of sample was applied to edge between coverslip and the hemocytometer chamber for counting cells. Cells were counted using microscope at 10× objective and snapshot of the field was taken. Live cells (unstained) that did not take the stain were considered. Dead cells were stained [22]. Cell survivability or viability was counted using formula [23] (Supplementary Figure S1).

$$\text{Viable cells }=\frac{\mathrm{Number}\;\mathrm{of}\;\mathrm{viable}\;\mathrm{cells}\;\mathrm{per}\;1\;\mathrm{ml}\;\mathrm{of}\;\mathrm{aliquot}}{\mathrm{Total}\;\mathrm{number}\;\mathrm{of}\;\mathrm{cells}\;\mathrm{per}\;1\;\mathrm{ml}\;\mathrm{of}\;\mathrm{aliquot}}\;*\;100$$

### Real-time polymerase chain reaction assay

SW982 cells were cultured in a 25 ml T-culture flask. After reaching 70–80% confluence, nicotine extract was added in serum-free media and incubated for 24 h. The effect of nicotine on inflammation was investigated by treatment with 10 ng/ml TNF-α on nicotine-pretreated cells for 1 h. Total RNA was isolated by using Tri-Xtract (G Biosciences) according to the manufacturer’s instructions and 0.8 µg of total RNA was used for cDNA preparation using cDNA Synthesis Kit (G Biosciences, U.S.A.). The transcribed cDNAs were mixed with respective primers and SyberGreen qPCR mix (G Biosciences, U.S.A.). Level of mRNA expression was evaluated using a Roche Light Cycler 480 II Real-Time PCR detection system. The data were normalized with β-actin internal control and analyzed quantitatively using 2^−ΔΔ*C*_T_^ formula [24]. Human-specific primers sequences are shown in Table 1.

**Table 1:** Synthesized primer sequences of cytokines and proteins required for qPCR

S.No.	Name of gene	Forward primer	Reverse primer
1	*IL-1β*	5′ AAACAGATGAAGTGCTCCTTCCAGG 3′	5′ TGGAGAACACCACTTGTTGCTCCA 3′
2	*IL-6*	5′ GGTACATCCTCGACGGCATCT 3′	5′ GTGCCTCTTTGCTGCTTTCAC 3′
3	*TNF-α*	5′ CCCCAGGGACCTCTCTCTAATC 3′	5′ GGTTTGCTACAACATGGGCTACA 3′
4	*TRAF-2*	5′ ACCAGCCCAGTCCTCAGATTTCAGA 3′	5′ CTAGGAATGCTCCCTTCTCTCTCCAG 3′
5	*β-actin*	5′ CATCCGCAAAGACCTGTACG 3′	5′ CCTGCTTGCTGATCCACATC 3′
6.	*P50*	5′ TCCACAAGGCAGCAAATAGA 3′	5′ GGGGCATTTTGTTGAGAG TT 3′
7.	*P65*	5′ GAAGAAGAGTCCTTTCAGCG 3′	5′ GGGAGGACGTAAAGGGATAG 3′

### Total protein extraction and Western blotting

Human synovial fibroblasts (SW982) were cultured as mentioned above until they reached 70–80% confluence. Pro-inflammatory cytokine responses were studied in serum-free medium. Cells were treated with nicotine, 24 h before the human recombinant TNF-α (10 ng/ml) induction, for 10 min. The cell lysate was extracted using ice-cold radioimmunoprecipitation assay (RIPA)-cell lysis buffer that contained protease and phosphatase inhibitors. The protein concentration was estimated by Bradford protein assay, separated on 12% SDS/PAGE, and electrotransferred using a Trans-Blot Semi-dry transfer unit (Bio-Rad, U.S.A.). The membranes were blocked for 4 h at RT ith 5% bovine serum albumin (Sigma–Aldrich, U.S.A.) followed by overnight incubation at 4°C with diluted (1:8000) primary antibodies (P65, pP65) along with GAPDH as loading control and IkBα (CST#4812) with tubulin (Santa Cruz, U.S.A.) as loading control. The blots were then washed and incubated for 1 h with diluted (1:10000) horseradish peroxidase (HRP) conjugated anti-mouse for phosphorylated and non-phosphorylated pP65 and for IkBα anti-rabbit secondary antibody (Jackson, U.S.A.) was used. The blots were then developed using enhanced chemiluminescence (ECL) (Thermo Scientific, Pierce, U.S.A.), and scanned by ChemiDoc™ MP Imaging system (Bio-Rad, U.S.A.) [25].

### Statistical analysis

To calculate the significant difference and graphing, GraphPad Prism 9 Software (U.S.A.) was used. Groups were analyzed by analysis of variance (ANOVA) with mean ± S.D. value. Only statistically significant (*P*≤0.05) values were considered.

## Results

### Quantitative HPLC analysis of nicotine extracted from *Brassica oleracea botrytis*

**Figure 1:**
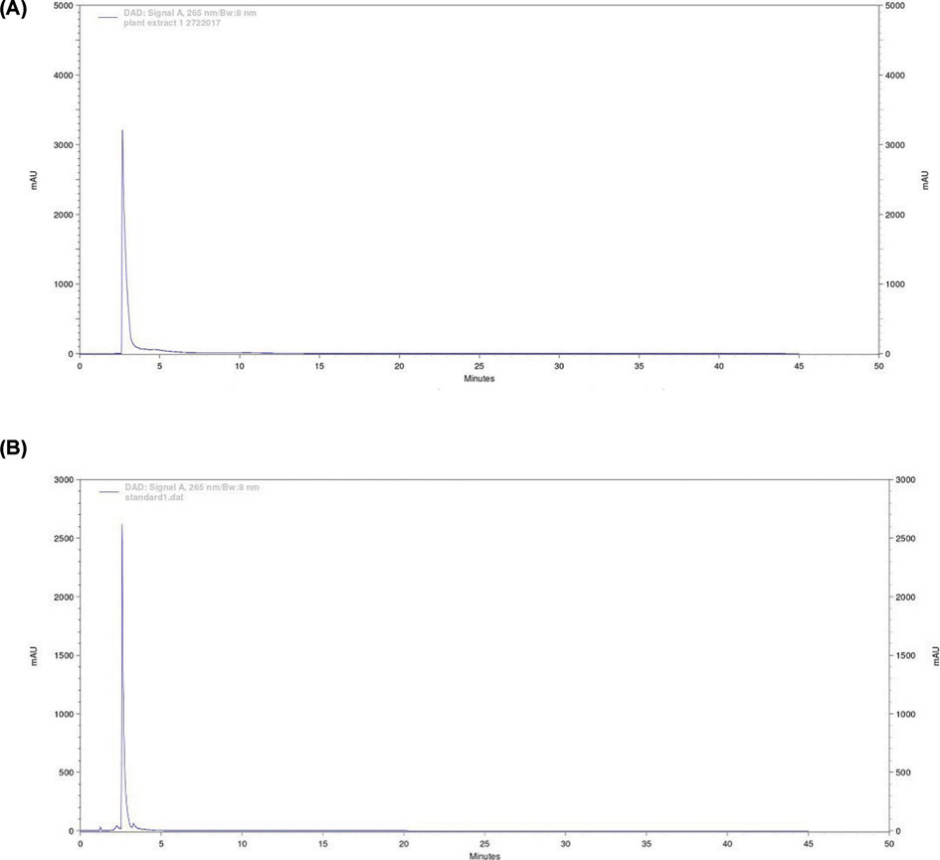
Typical HPLC chromatograms (**A**) HPLC chromatographic profile of nicotine isolated from *B. oleracea.* (**B**) HPLC chromatographic profile of nicotine standard prepared from tobacco leaves.

Extraction of nicotine from *Brassica oleracea botrytis* was validated by HPLC method using calibration curve. Five different concentrations (ranging from 10 to 50 mg/ml) of standard solution of nicotine was used. Calibration curve was constructed by plotting peak areas against analyte concentrations. The linearity was assessed by calculating the slope, y-intercept, and coefficient of determination (r^2^) using least squares regression [26]. The slope (0.6), y-intercept (70), and correlation coefficient (r^2^) (0.8823) were obtained from regression analysis equation (y = 0.6 × −7E). The calibration curve was linear in the tested concentration ranges. We obtained correlation coefficient 0.8823, indicating a good degree of correlation and good linearity of method. Quantitative results of HPLC showed well-resolved nicotine peaks isolated from *B. oleracea* (with peak area 828979.194) and standard (with peak area 110186530), with retention time 2.67 min (Figure 1A,B). The concentration of nicotine from the methanol extracts was quantified as 2 mg/ml.

### Molecular docking analysis of nicotine and TNFR interaction, active site prediction to discover drug-likeliness properties

**Figure 2:**
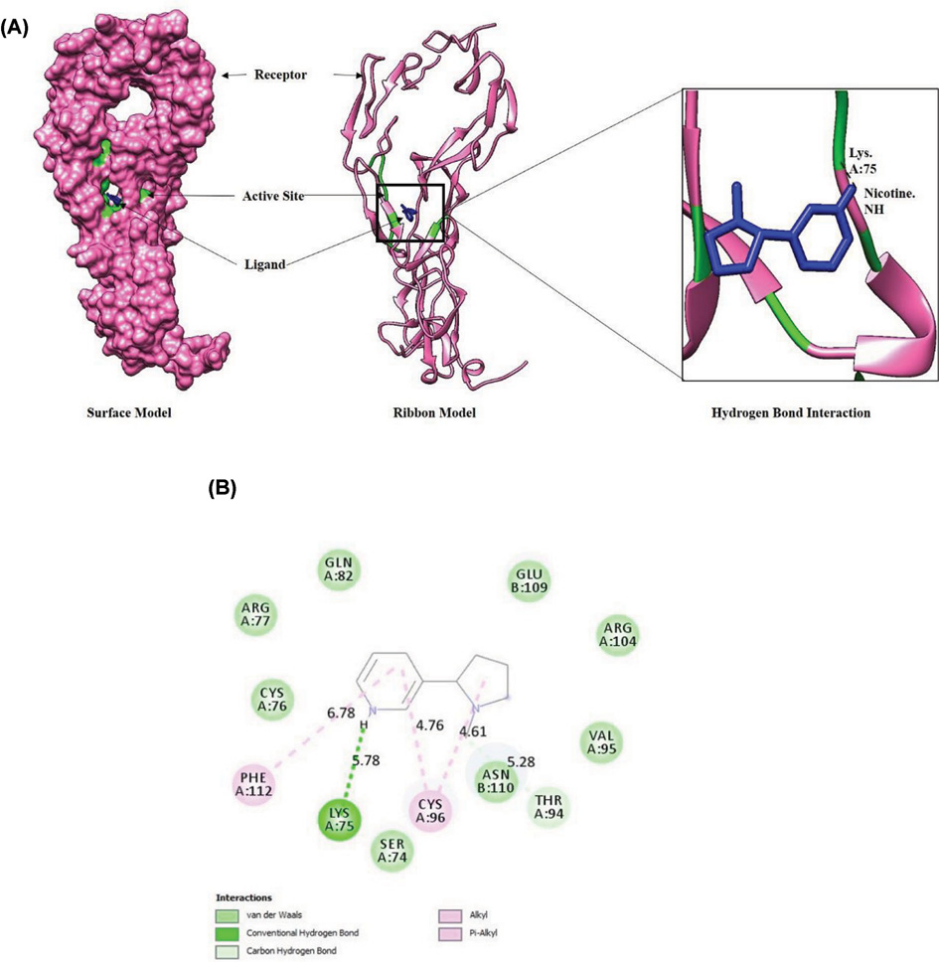
*In-silico* analysis (**A**) Docking study between nicotine as ligand and TNFR as receptor through Swissdock. (**B**) 2D interaction between ligand and receptor showing different types of bonds and bond lengths.

Protein-ligand docking of nicotine and TNFR was analyzed using Discovery Studio Visualizer and UCSF Chimera. The docked cluster has full fitness with energy −1757.92 kcal/mol and ΔG −5.91 kcal/mol. The analysis of docked molecules of ligand and receptor showed prominent results. The active site of TNFR was found to be occupied by amino acids, Phe^60^, Leu^71^, Ser^72^, Cys^73^, Ser^74^, Lys^75^, Arg^77^, Gln^82^, Cys^96^, Asn^110^, and Leu^111^ of chain-A that interacts with the pro-inflammatory ligand, TNFα [27]. Nicotine also interacts with the active sites of TNFR (Figure 2A,B). Lys^75^ of TNFR form hydrogen bond with the nicotine, suggesting the inhibition of TNFα to bind with TNFR.

**Figure 3:**
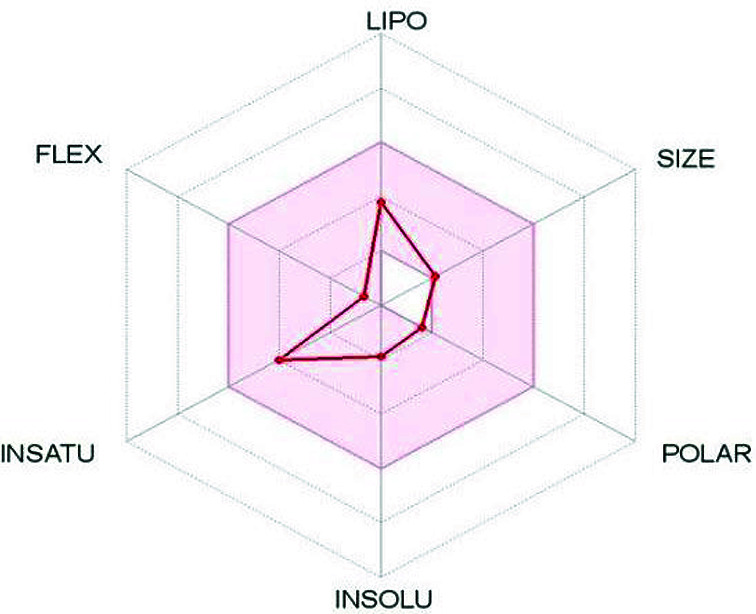
Bioavailability Radar showing and signifying the drug-likeliness of nicotine

The physicochemical and pharmacokinetic properties of the compound (nicotine) are shown in Table 2. During prediction, Bioavailability Radar was displayed for six physicochemical properties: lipophilicity, size, polarity, solubility, flexibility and saturation (Figure 3). The pink area, the radar plot of the molecule, was considered as drug-likeness, since it is following Lipinski’s rule of five. As per Lipinski’s rule of five, a chemical compound to be orally active in human should follow a minimum of three criteria of the following: (a) molecular weight ≤ 500, (b) XLOGP3 < 3.5, (c) hydrogen bond acceptor ≤ 10, (d) hydrogen bond donor ≤ 5 and molar refractivity = 40–130. In our study nicotine is following RO5 rules, suggesting that it could be suitable for oral administration. CYP450 and its isoforms belong to hemoprotein family, play crucial role in drug metabolism and clearance. Inhibition of the isoforms leads to lower clearance and accumulation of drug or its metabolism. Gastrointestinal (GI) absorption indicates the capacity of drug absorption and transmission into the bloodstream. The compound having non-inhibition CYP450, and high GI absorption properties expresses that the compound has a good capacity for metabolization and absorption [17]. The possible biological activity of all the compounds was retrieved by PASS server. It also predicts the set of pharmacological effects, mechanisms of action and specific toxicities, that might be exhibited by a compound in its interaction with biological entities [28]. The compound exhibited wide range of biological activities such as (S)-6-hydroxynicotine oxidase inhibitor, nAChR antagonist, antineurotic, respiratory analeptic etc. analyzed and the properties that could involve in the disease regulation were taken into consideration. It was found that nicotine had potential to act as an inhibitor to various oxidative stress molecules that could otherwise lead to chronic inflammation (Table 3).

**Table 2:** Physiochemical and pharmacokinetic properties of nicotine

**Physiochemical properties**	
Formula	C_10_H_14_N_2_
Molecular weight	162.23 g/mol
Number of heavy atoms	12
Number of aromatic heavy atoms	6
Fraction Csp3	0.50
Number of rotatable bonds	1
Number of H-bond acceptors	2
Number of H-bond donors	0
Molar refractivity	53.13
Lipophilicity	
Log Po/w (XLOGP3)	1.17
Water solubility	
Log S (ESOL)	−1.89
Solubility	2.10e + 00 mg/ml; 1.30e-02 mol/l, very soluble
**Pharmacokinetics**	
GI absorption	High
BBB permeant	Yes
P-gp substrate	No
CYP1A2 inhibitor	No
CYP2C19 inhibitor	No
CYP2C9 inhibitor	No
CYP2D6 inhibitor	No
CYP3A4 inhibitor	No

**Table 3:** Biological activities of nicotine

Pa	Pi	Activity
0,964	0,001	(S)-6-hydroxynicotine oxidase inhibitor
0,954	0,001	Nicotinic α6β3β4α5 receptor antagonist
0,946	0,002	Nicotinic α2β2 receptor antagonist
0,931	0,001	(R)-6-hydroxynicotine oxidase inhibitor
0,932	0,001	CYP2A8 substrate
0,898	0,003	Nicotinic α4β4 receptor agonist
0,881	0,006	Antineurotic
0,879	0,008	Nootropic
0,781	0,004	CYP2E1 inhibitor
0,766	0,002	Acetylcholine nicotinic antagonist
0,767	0,010	Respiratory analeptic
0,759	0,004	Oxygen scavenger
0,748	0,007	CYP2A6 substrate
0,729	0,001	Neuronal nicotinic receptor antagonist
0,730	0,005	Anti-hypoxic
0,732	0,009	CYP2A substrate
0,742	0,025	Glycosylphosphatidylinositol phospholipase D inhibitor
0,716	0,003	Cholinergic antagonist
0,715	0,003	Acetylcholine antagonist
0,719	0,023	5-Hydroxy tryptamine release stimulants
0,717	0,032	CYP2H substrate

It is reported that nicotine compound affects various biological functions, regulation of hormone secretion and enzyme activities ranging from gene expression [29]. The gene expression of the compound was evaluated using DIGEP-Pred. It showed a plethora of genes, involved in various kinds of biological activities that has been regulated positively/negatively by the nicotine compound (Table 4). It has been found that the gene expression that got changed after nicotine treatment were namely IVL, PRL, PLAT, GH1, CYP1A2, CYP1A1 and PROC genes. Among them IVL and PRL genes were found down-regulated, rest of the genes were found to be up-regulated by nicotine treatment and PROC (Protein C, Inactivator of Coagulation Factors Va and VIIIa) was found to be involved in controlling inflammation and promoting blood clot formation [30].

**Table 4:** Cellular gene expressions induced by nicotine

Pa	Pi	Regulation
0.83	0.008	IVL ↓
0.721	0.004	PRL ↓
0.969	0.002	PLAT ↑
0.862	0.01	GH1 ↑
0.827	0.009	CYP1A2 ↑
0.565	0.039	CYP1A1 ↑
0.504	0.085	PROC ↑

### Cell viability, pro-inflammatory cytokines mRNA expression and anti-inflammatory effects of nicotine

**Figure 4:**
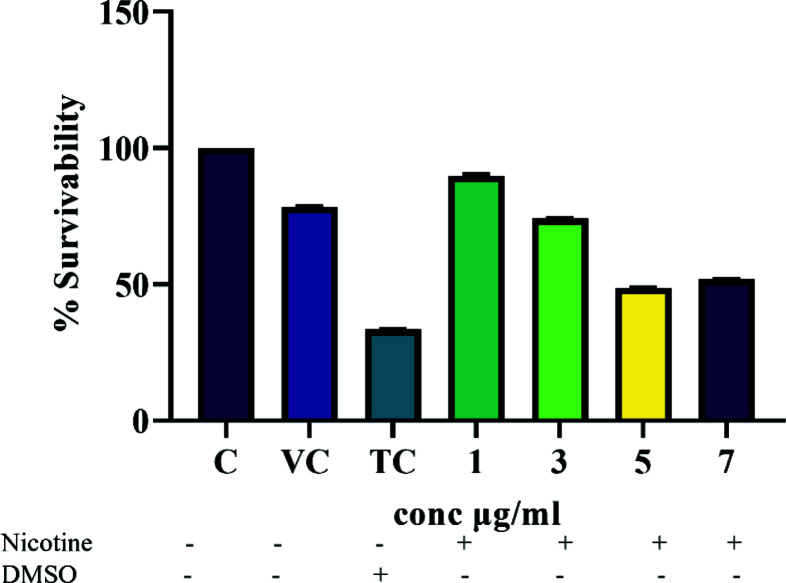
Cell viability test of nicotine extract on SW982 performed by MTT DMSO was taken as toxic control (TC), vehicle control (VC) and 1, 3, 5, 7 µg/ml concentration of nicotine.

**Figure 5:**
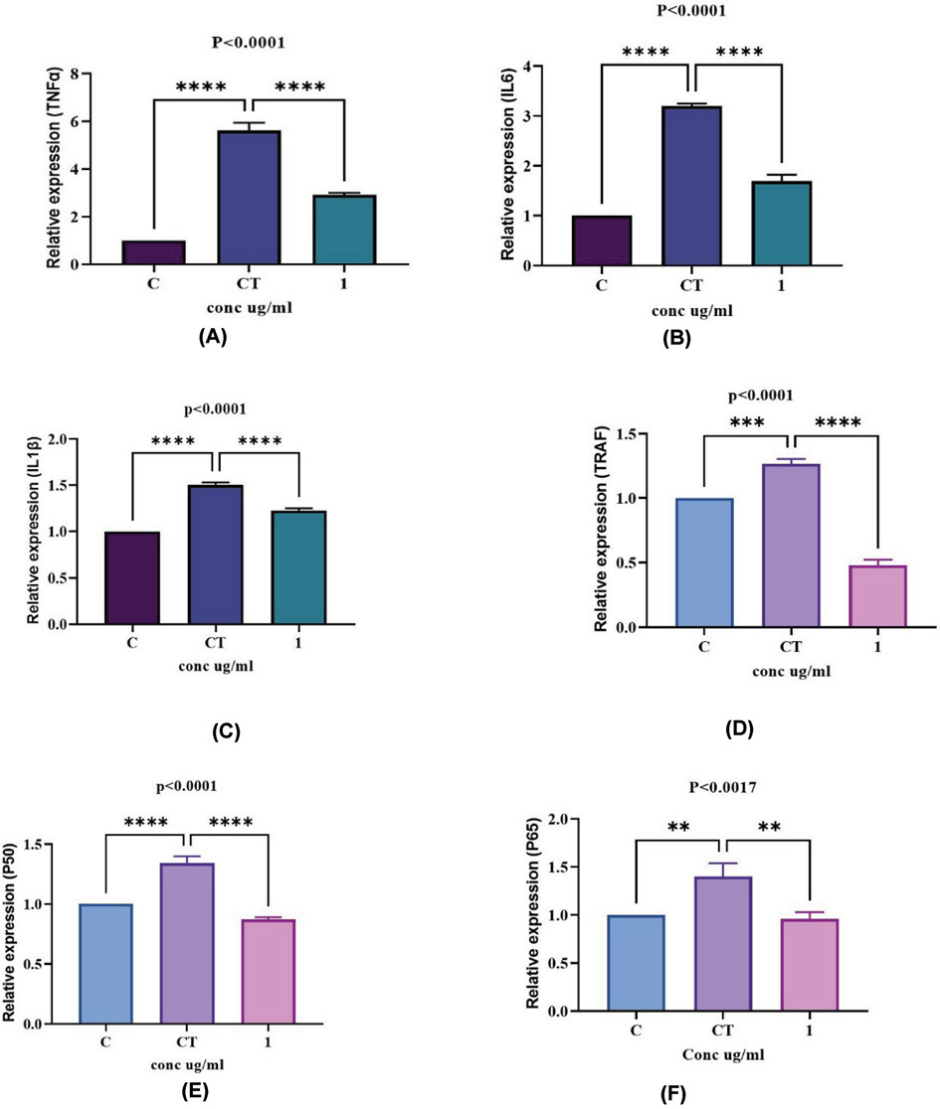
Real-time polymerase chain reaction assay of pro-inflammatory cytokines and proteins Fold induction of TNF-α, TRAF, IL-6, IL-1β mRNA expression in SW982 when treated with TNF-α at 10 ng/ml or nicotine at 1 µg/ml or co-treated with TNF-α and nicotine compared with respective controls. C = Control cells without TNF-α and extract, CT = cells with TNF-α 10 ng/ml for 1 h, 1 = cells with extract at 1 µg/ml concentration for 24 h with TNF-α at 10 ng/ml for 1 h. (**A**) Fold induction of TNF-α gene expression in SW982 cells. (**B**) Fold induction of IL-6 gene expression in SW982 cells. (**C**) Fold induction of IL-1β gene expression in SW982 cells. (**D**) Fold induction of TRAF gene expression in SW982 cells. (**E**) Fold induction of P50 gene expression in SW982 cells. (**F**) Fold induction of P65 gene expression in SW982 cells. Values are presented as the mean ± SEM (*n*=3). **** = <0.0001,*** = <0.001 ** = <0.01 versus normal control or TNF-α treatment (*) by one-way ANOVA.

We measured cell viability by MTT assay and by Trypan Blue staining method using SW982 cells. The survivability of cells were found to be maximum at 1 µg/ml (optimized concentration) of nicotine extract (Figure 4). The nicotine effectiveness was studied and analyzed via gene expression profile of pro-inflammatory cytokine (IL-1β, IL-6, TNF-α), mediator proteins (TRAF2, P50 (NF-κB1)) and P65 (RelA) that are associated with NF-kB inflammatory pathway. Interestingly, the nicotine treatment showed significantly decreased level of mRNA expression of TNF-α (Figure 5A), IL-6 (Figure 5B), IL-1β (Figure 5C), TRAF2 (Figure 5D), P50 (Figure 5E) and P65 (Figure 5F) compared with control cells. The cells without TNF-α treatment were considered as control cells. The results thus suggested that nicotine could suppress the mRNA expression of pro-inflammatory cytokines that were induced by TNF-α.

### Expression of pro-inflammatory proteins via Western blot analysis

**Figure 6:**
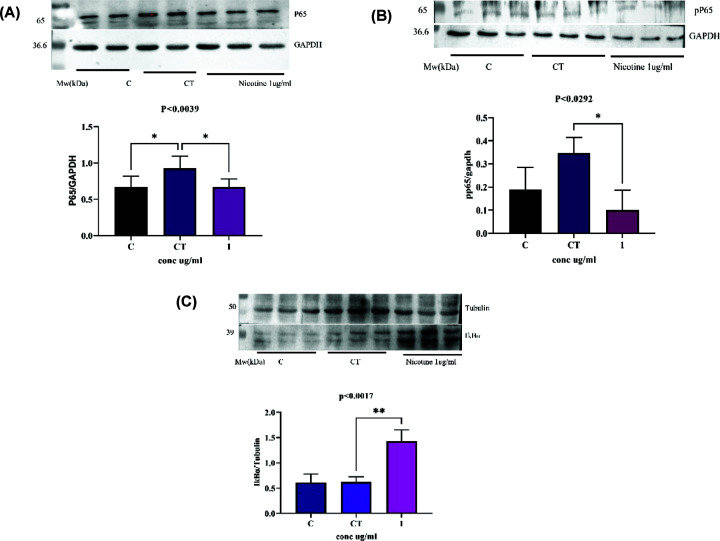
Western blot analysis of P65, pP65 and IkBα in SW982 cells The expression levels of P65, pP65 and IkBα were observed in SW982 under TNF-α-induced conditions; 10 ng/ml induction for 10 min in SW982 cells pretreated with or without 1 µg/ml nicotine. (**A**) The Western blot of P65, (**B**) pP65 and (**C**) IkBα. The band densities indicate significant difference between control, with or without TNF-α and nicotine treatments. The bar graphs represent P65, pP65 and IkBα band densities relative to the total form of each GAPDH and tubulin band intensity determined by densitometric analysis. Values are presented as the mean ± SEM. The significance levels indicate * = *P*<0.05; versus control 0 min (C) with TNF-α induction for 10 min (CT) and ** = *P*<0.01 with 24-h pretreatment with nicotine measured using one-way ANOVA. Abbreviations: C, control cells without TNF and nicotine, CT, cells with TNF-α induction for 10 min; nicotine, cells with 24-h pretreated nicotine with TNF-α induction for 10 min.

Human synovial fibroblasts (SW982) showed a significant expression upon TNF-α exposure at 10 ng/ml for 10 min compared with the control cells (un-induced). The fold induction of P65 and pP65 expression resulted into significantly lower expression with *P*<0.0039 (Figure 6A) and *P*<0.0292 (Figure 6B), respectively, in nicotine-pretreated cells while the expression of IkBα was increased with *P*<0.0017 (Figure 6C). For normalization, GAPDH was used for p65 and tubulin for IkBα as loading control. The results thus suggested that nicotine is effectively reducing p65 and increasing IkBα expression, leading to lower the incidence of inflammatory conditions of RA human synoviocytes.

## Discussion

RA is systemic, autoimmune, inflammatory disease affecting majorly joints and their related tissues [1]. Synovial inflammation and autoantibodies generation are the characteristic features of the disease [4]. Available drugs can overcome the disease severity but with severe side effects, the herbal and nutrient-rich food world is giving hope to cover this situation. Out of several different nutrient-rich foods, *Brassica* vegetables are highly recommended due to their high nutrition, richness in vitamins and various properties such as antioxidant, anticancer and anti-inflammation [31].

At molecular level, there are several biomolecules derived from *B. oleracea* such as exogenous microRNA, contributing to gene regulation of distantly related organisms by increasing their concentrations in blood/plasma/serum or in organs. *In-vivo* studies reported its regulatory role by increasing the level of *B. oleracea* miRNA [32], indicating that the use of naturally occurring food-derived products/regulatory compounds/molecules from *B. oleracea* is beneficial in minimizing the disease symptoms. Therefore, we were interested to identify dietary components from *B. oleracea* with a belief and hope that the identified components may resolve the inflammation occurrence associated with RA without any side effect(s).

Nicotine, a psychoactive component/alkaloid, produced by tobacco plants acts on naturally occurring neurotransmitter acetylcholine, nicotinic acetylcholine receptors that are present on many cells and have an immunological effect [33,34]. Several studies have reported nicotine to serve as an anti-inflammatory compound, cause lower risk of inflammatory diseases such as ulcerative colitis, obesity, that are considered low-grade inflammation [35]. In Alzheimer’s disease, Crohn’s disease and Parkinson’s disease, nicotine plays a protective role through activation of cholinergic anti-inflammatory response pathway in targeted cells [36]. During inflammation, TNF-α released by macrophages, inhibited by the cholinergic anti-inflammatory pathway [37]. The physiological interaction between neuronal type α7 nicotinic acetylcholine receptor (α7AChR) and its agonist directly activates the cholinergic anti-inflammatory pathway. The interaction between α7nAChR and nicotine suppresses the phosphorylation of IkB, which is an inhibitor of NF-kB [38,39].

Reports also show that pretreatment of nicotine with a low dose inhibits the production of pro-inflammatory mediators such as TNF-α, macrophage inflammatory protein (MIP)-1α and prostaglandin E-2 [39]. Nicotine isolated from *B. oleracea* is S-isomer of nicotine, abundant in natural sources. Since the commercially available nicotine is different from isolated nicotine [12], we obtained standard graph using commercially available tobacco leaves, to match the profile of isolated nicotine. Our aim was to identify anti-inflammatory effect of nicotine that is present naturally in *B. oleracea* that may be useful to reduce inflammation in RA condition. Earlier it has been reported that the natural extract is highly effective and has many beneficial roles without side effects [13]. Commercially synthesized nicotine has many side effects such as nausea, dizziness, light headaches, sleep disturbance, contact dermatitis etc., and thus it is difficult to predict synthesized nicotine for its clinical importance [40]. In *B. oleracea*, the nicotine content is very less compared with other natural sources, and it is consumed on daily basis all over the world, indicating it to be a nontoxic natural product [41]. In cigarettes or in nicotine-containing products, nicotine interacts with other harmful compounds such as hydrogen cyanide, ammonia and aromatic amines [42], but such interaction is absent from *B. oleracea*. The other nicotine-containing vegetables are potato, tomato and other nightshade family, but they also contain solanine as a toxic alkaloid [41]. *B. oleracea*, source of nicotine in the present study nullifies all toxic effect on mammals and hence can be more useful as effectiveness, quality and safety is highly dependent on the source of the isolated compound. A low dose of nicotine has many pharmacological effects, but a higher dose of nicotine can be toxic in mammals (50–100 mg) [43]. Thus, we performed *in-vitro* experiments using lower dose of nicotine concentrations ranging from 0.1 to 1.5 µg/ml and observed that at higher concentrations cell viability gets decreased. At 1 µg/ml concentration, nicotine was showing its activity without affecting cell viability and below 1 µg/ml, nicotine was not showing its optimum effect (Figure 4). Hence, 1 µg/ml nicotine concentration was used for further experiments. In RA pathogenesis, the anti-inflammatory properties of nicotine have been poorly demonstrated. Nicotine affects inflammatory pathways (NF-kB pathway) due to its immunosuppressive or anti-inflammatory properties, inhibits phosphorylation of I-kB, resulting into lower production of inflammatory cytokines [39]. Nicotine is also associated with humoral as well as cell-mediated immunity [44–46]. The exposure induces regulatory T cells [47] that affects the survival and development of B lymphocytes through activation of nicotinic acetylcholine receptors [48]. α-7 nicotinic receptor is a type of acetylcholine receptor present on various immune cells like macrophages, dendritic cells, monocytes, B and T lymphocytes and fibroblasts residing in the inflamed synovial tissue of RA [49].

In our findings, the diet component and nicotine were studied in correlation, wherein nicotine was extracted from a natural dietary source (*B. oleracea*), and its anti-inflammatory effects were evaluated. Report shows that the interaction between TNFα and TNFR causes increase in inflammation via cytokines production that are involved in autoimmune diseases like inflammatory bowel disease, RA, systemic sclerosis and diabetes [50]. Earlier, various studies were conducted to identify potential inhibitors of TNFα/TNFR to reduce inflammation [51]. As we were interested to find out the reasons for reduction in inflammation, interaction studies were performed that showed direct interaction of nicotine with TNFR active sites, gave us a clue that nicotine may be a potential activator of cholinergic anti-inflammatory pathway. We, therefore speculated that probably a small molecule is binding directly to TNFα or TNFR, inhibiting/modulating their interaction and thereby acting as a potential inhibitor of the pro-inflammation mechanism. To confirm our belief, we explored more results via *in-silico* experiments. Our results confirmed that the active-binding sites of TNFR and TNF α are involved in the inflammatory responses (Figure 2). Nicotine also showed higher binding affinity with the receptor suggesting that dietary compounds of *B. oleracea* may inhibit the interaction of cytokines with its receptors.

Further, our interest was to see the anti-inflammatory role of nicotine and hence investigation in synovitis-like model was carried out. TNFα is a prominent cytokine of RA, hence used to mimic the RA condition using human synovial sarcoma cell SW982. Nicotine (1 µg/ml) was found to be effective (Figure 4) in reducing mRNA expression of pro-inflammatory cytokines level and mediator proteins of the NF-kB pathway (Figure 5), decreased the protein expression of P65 (Figure 6A) and its phosphorylated form, pP65, further (Figure 6B) and increased the level of IkBα (Figure 6C). Previously, it has been demonstrated that TNF-α binds to TNFR, subsequently activates multiple kinases to initiate activation of IκB kinase (IKK). Also, phosphorylation of IκB at specific amino acids by IKK activates the proteasomal degradation of IκB which in turn releases dimers of NF-κB (p50/65) and translocate into cell nucleus [52,53]. It binds with enhancer element of target genes and initiates the expression of pro-inflammatory cytokines (IL6, IL-1β) and protein (P65) [54]. Nicotine extracted from natural source *B. oleracea* thus acted as a potent anti-inflammatory compound, indicating the efficacy of nicotine as an anti-inflammatory compound at lower doses. Our findings showed that nicotine purified from natural source may be used as therapeutic agent for arthritis.

## Conclusion

The study proposed that anti-inflammatory effect of nicotine is present in *Brassica* vegetables at lower concentrations. Nicotine effectively reduces the levels of proinflammatory cytokines and proteins and reduces inflammation. The investigations had certain limitations such as the diet sample that needed more analyses to determine the optimum consumption of nicotine. Apart from cytokines, there may be other protein moieties that may be playing a key role in the clinical development of RA. Nicotine in conjugation with other bioactive molecules could be studied for their therapeutic potential and clinical significance. Further investigations of nicotine isolated from various such sources would be required to assess their potential as therapeutic agents that are usually associated with nicotine.

## Supplementary Material

Supplementary Figure S1

## Data Availability

Data availability and data supporting the findings of the present study are available from Sagarika Biswas (corresponding author) on request.

## Abbreviations

ADME: absorption, distribution, metabolism and excretion
DIGEP: drug-induced Gene Expression Profile
GI: gastrointestinal
HPLC: high profile/phase liquid chromatography
IKK: IκB kinase
NF-kB: nuclear factor-κ B
PROC: Protein C, Inactivator of Coagulation Factors Va and VIIIa
RA: rheumatoid arthritis
RO5: Lipinski’s rule of five
RT: room temperature
SMILES: simplified molecular input line-entry system
TNFα: tumor necrosis factor α
TRAF2: TNF receptor-associated factor 2
