# Supplementary material for: Anti-inflammatory activity of nicotine isolated from Brassica oleracea in rheumatoid arthritis
Source: Biosci Rep. 2022 Apr 21;42(4):BSR20211392. doi: 10.1042/BSR20211392 (PMC9069442; doi:10.1042/BSR20211392)

Supplementary figure 1: Cell viability test of nicotine extract on SW982 performed by Trypan blue staining. **A)** Trypan staining cells showing in hemocytometer **B)** Cell survivability graph calculated by formula given below:

$$\text{Viable cells} = \frac{Number\ of\ viable\ cells\ per\ 1ml\ of\ aliquot}{Total\ number\ of\ cells\ \ per\ 1ml\ of\ aliquot} * 100$$

A)

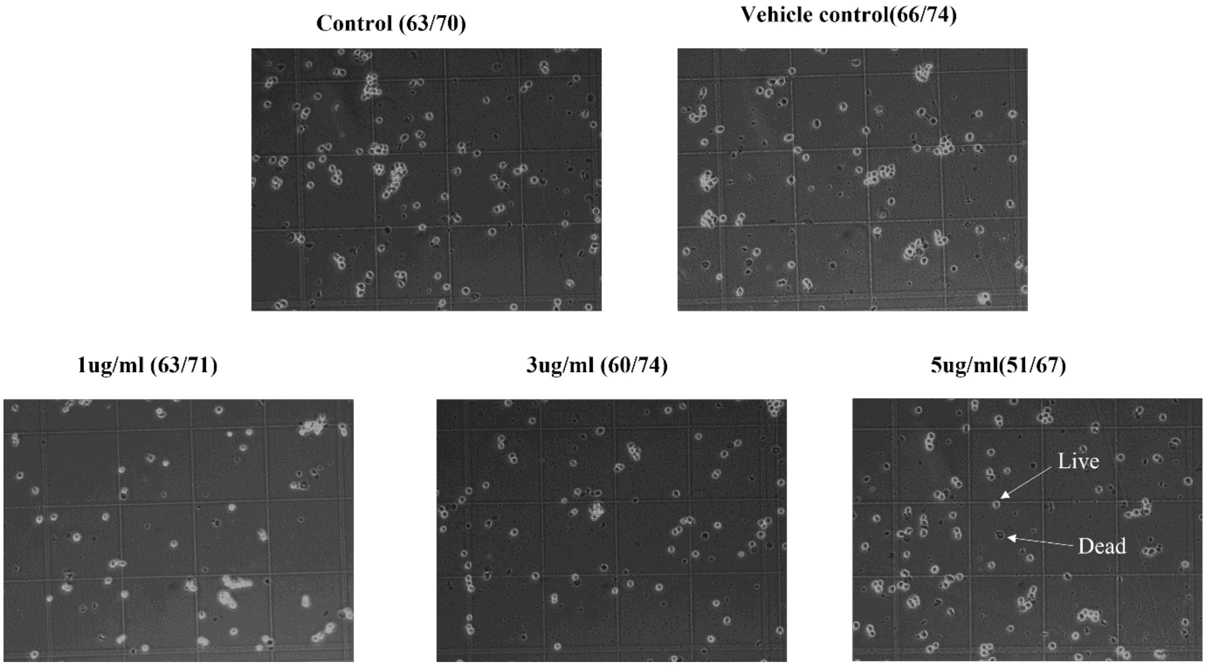

B)

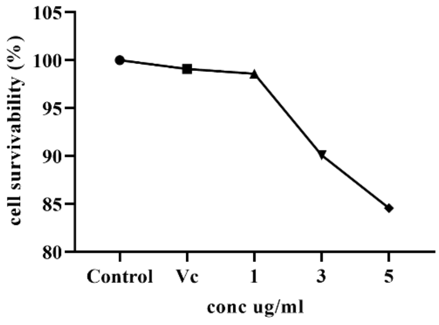

Supplement: Supplementary Figure S1 [file BSR-2021-1392_supp.pdf]
